# Antibody response durability following three-dose coronavirus disease 2019 vaccination in people with HIV receiving suppressive antiretroviral therapy

**DOI:** 10.1097/QAD.0000000000003469

**Published:** 2022-12-22

**Authors:** Hope R. Lapointe, Francis Mwimanzi, Peter K. Cheung, Yurou Sang, Fatima Yaseen, Sarah Speckmaier, Evan Barad, Nadia Moran-Garcia, Sneha Datwani, Maggie C. Duncan, Rebecca Kalikawe, Siobhan Ennis, Landon Young, Bruce Ganase, F. Harrison Omondi, Gisele Umviligihozo, Winnie Dong, Junine Toy, Paul Sereda, Laura Burns, Cecilia T. Costiniuk, Curtis Cooper, Aslam H. Anis, Victor Leung, Daniel Holmes, Mari L. DeMarco, Janet Simons, Malcolm Hedgcock, Natalie Prystajecky, Christopher F. Lowe, Marc G. Romney, Rolando Barrios, Silvia Guillemi, Chanson J. Brumme, Julio S.G. Montaner, Mark Hull, Marianne Harris, Masahiro Niikura, Mark A. Brockman, Zabrina L. Brumme

**Affiliations:** a British Columbia Centre for Excellence in HIV/AIDS, Vancouver; b Faculty of Health Sciences; c Department of Molecular Biology and Biochemistry, Simon Fraser University, Burnaby; d Division of Medical Microbiology and Virology; e AIDS Research Program, St. Paul's Hospital; f Department of Pathology and Laboratory Medicine, Providence Healthcare, Vancouver; g Division of Infectious Diseases and Chronic Viral Illness Service, McGill University Health Centre and Research Institute of the McGill University Health Centre, Montreal, Quebec; h Department of Medicine, University of Ottawa; i Ottawa Hospital Research Institute, Ottawa; j School of Population and Public Health; k CIHR Canadian HIV Trials Network, University of British Columbia; l Centre for Health Evaluation and Outcome Sciences, Vancouver; m Department of Pathology and Laboratory Medicine, University of British Columbia; n Spectrum Health; o British Columbia Centre for Disease Control Public Health Laboratory, Vancouver; p Department of Family Practice, Faculty of Medicine, University of British Columbia; q Department of Medicine, University of British Columbia, Vancouver, Canada.

**Keywords:** antibody, coronavirus disease 2019, HIV, humoral immunity, hybrid immunity, Omicron BA.1, Omicron BA.5, third dose, vaccines, viral neutralization

## Abstract

**Background:**

Limited data exist regarding longer term antibody responses following three-dose coronavirus disease 2019 (COVID-19) vaccination, and the impact of a first SARS-CoV-2 infection during this time, in people with HIV (PWH) receiving suppressive antiretroviral therapy (ART). We quantified wild-type-specific, Omicron BA.1-specific and Omicron BA.5-specific responses up to 6 months post-third dose in 64 PWH and 117 controls who remained COVID-19-naive or experienced their first SARS-CoV-2 infection during this time.

**Design:**

Longitudinal observational cohort.

**Methods:**

We quantified wild-type-specific and Omicron-specific anti-Spike receptor-binding domain IgG concentrations, ACE2 displacement activities and live virus neutralization at 1, 3 and 6 months post-third vaccine dose.

**Results:**

Third doses boosted all antibody measures above two-dose levels, but BA.1-specific responses remained significantly lower than wild-type-specific ones, with BA.5-specific responses lower still. Serum IgG concentrations declined at similar rates in COVID-19-naive PWH and controls post-third dose (median wild-type-specific and BA.1-specific half-lives were between 66 and 74 days for both groups). Antibody function also declined significantly yet comparably between groups: 6 months post-third dose, BA.1-specific neutralization was undetectable in more than 80% of COVID-19 naive PWH and more than 90% of controls. Breakthrough SARS-CoV-2 infection boosted antibody concentrations and function significantly above vaccine-induced levels in both PWH and controls, though BA.5-specific neutralization remained significantly poorer than BA.1 even post-breakthrough.

**Conclusion:**

Following three-dose COVID-19 vaccination, antibody response durability in PWH receiving ART is comparable with controls. PWH also mounted strong responses to breakthrough infection. Due to temporal response declines, however, COVID-19-naive individuals, regardless of HIV status, would benefit from a fourth dose within 6 months of their third.

## Introduction

In British Columbia (BC), Canada, third doses of coronavirus disease 2019 (COVID-19) monovalent mRNA vaccines were introduced in November 2021, initially to individuals at risk of severe COVID-19 outcomes, including some people with HIV (PWH). Whether offered as part of a primary vaccine series or a ‘booster’, third doses help to maintain systemic immunity and enhance protection against infection by viral variants [1–4]. Despite being effective at preventing severe disease because of SARS-CoV-2, third doses provide limited protection against transmission of Omicron subvariants, including BA.1 and BA.5 [5–10], which were estimated to have infected more than 60% of Canadians by August 2022, despite approximately 50% uptake of third vaccine doses [11–13].

Longitudinal monitoring of immune responses post-third dose in PWH is critical to inform the timing of future immunizations in this group. Though some data are available on initial immunogenicity to third COVID-19 vaccine doses in PWH [14–16], no studies to our knowledge have assessed the longer term durability of post-third dose responses in this population. Furthermore, despite the high incidence of first-time SARS-CoV-2 infections after three vaccine doses, no studies to our knowledge have examined the impact of such infections on responses in PWH. Here, we extend prior observations from our cohort [14,17] by quantifying wild-type-specific, Omicron BA.1-specific and BA.5-specific responses up to 6 months post-third vaccine dose in 64 PWH and 117 controls who either remained COVID-19-naive or experienced their first (presumably Omicron [18]) SARS-CoV-2 infection, during this period.

## Methods

### Participants

Our cohort was described previously [14]. The present study included 64 PWH and 117 controls who remained COVID-19-naive until at least 1 month post-third vaccine dose. Breakthrough SARS-CoV-2 infections were identified through self-reported PCR and/or rapid-antigen test results and the presence of serum antibodies against Nucleocapsid (N) using the Elecsys Anti-SARS-CoV-2 assay (Roche Diagnostics, Laval, Quebec, Canada).

### Ethics approval

This study was approved by the University of British Columbia/Providence Healthcare and Simon Fraser University Research Ethics Boards. All participants provided written informed consent.

### Antibody assays

Assays were performed as previously described [14,19]. IgG-binding antibodies in serum were measured against the SARS-CoV-2 Spike Receptor Binding Domain (RBD) using the V-plex SARS-CoV-2 (IgG) ELISA kit (Panel 22; Meso Scale Diagnostics, Rockville, Maryland, USA), which features wild-type and Omicron-BA.1 RBD antigens, on a Meso QuickPlex SQ120 instrument. Serum was diluted 1 : 10000 and reported in WHO International Standard Binding Antibody Units (BAU)/ml using the manufacturer-supplied conversions. Surrogate virus neutralization activity [20] in serum was measured by competition ELISA using the same kit [Panel 22; V-plex SARS-CoV-2 (ACE2)] to measure blockade of the RBD-ACE2 receptor interaction. Sera were diluted 1 : 40 and results reported as % ACE2 displacement. Virus neutralizing activity in plasma was assessed using live wild-type (USA-WA1/2020; BEI Resources, Manassas, Virginia, USA), and two local isolates identified as Omicron BA.1 (GISAID Accession# EPI_ISL_9805779) and Omicron BA.5 (GISAID Accession# EPI_ISL_15226696) on VeroE6-TMPRSS2 (JCRB-1819) target cells [19]. Virus stocks were diluted to 50 TCID_50_/200 μl in the presence of serial two-fold plasma dilutions (1/20 to 1/2560) and added to target cells in triplicate. Viral cytopathic effects (CPE) were recorded 3 days post-infection. Neutralization was reported as the highest reciprocal dilution able to prevent CPE in all three wells. Partial or no neutralization at 1/20 dilution was considered below the limit of quantification (BLOQ) and coded as a reciprocal dilution of 10.

### Statistical analyses

Continuous variables were compared using the Mann–Whitney *U* test (unpaired data) or Wilcoxon test (paired data). Relationships between continuous variables were assessed using Spearman's correlation. In participants who remained COVID-19-naive, multiple linear regression was used to investigate the relationship between HIV infection and vaccine-induced immune measures using a confounder model that adjusted for variables that could influence vaccine responses or that differed in prevalence between groups. For Omicron-specific neutralization at 6 months post-third dose, multiple logistic regression was used because of the high proportion of results BLOQ. Included variables were: HIV infection (controls as reference group), age (per year), sex at birth (female as reference), ethnicity (nonwhite as reference), number of chronic conditions (per additional), dual ChAdOx1 as the initial regimen [mRNA or mixed (ChAdOx1/mRNA) regimen as the combined reference group] [14,17], third COVID-19 mRNA dose brand (BNT162b2 as reference) and the interval between second and third doses (per day). Plasma neutralization models also corrected for anticoagulant (ACD as reference). All tests were two-tailed, with *P* less than 0.05 considered statistically significant. Analyses were conducted using Prism v9.2.0 (GraphPad Software, San Diego, California, USA).

## Results

### Participant characteristics

Characteristics of the 64 PWH and 117 controls, all of whom remained COVID-19-naive until at least 1 month post-third dose, are shown in Table 1. All PWH had suppressed plasma HIV viremia on ART, median CD4^+^ T-cell counts of 645 [interquartile range (IQR) 473–958] cells/μl, and median nadir CD4^+^ T-cell counts of 225 (IQR 95–485) cells/μl, at enrolment. PWH were a median of 57 (IQR 42–65) years old and 90% men; controls were a median 47 (IQR 35–72) years old and 73% women. PWH had a higher proportion of white ethnicity (72%, compared with 55% in controls) and more chronic health conditions [median 1 (IQR 1–3) compared with 0 (IQR 0–1) in controls]. More PWH (10%) than controls (<1%) received two doses of the recombinant viral vector ChAdOx1 vaccine as their initial immunization series. All third doses were monovalent mRNA vaccines, either BNT162b2 (30 μg) or mRNA-1273 (50 or 100 μg). Most PWH (69%) and controls (62%) received mRNA-1273, where, per local guidelines, all adults aged at least 70 years were eligible for a 100 μg mRNA-1273 dose, as were PWH who met one or more of the following criteria: age at least 65 years, prior AIDS-defining illness, prior CD4^+^ T-cell count less than 200 cells/μl, prior CD4^+^ T-cell fraction 15% or less, any plasma HIV load greater than 50 copies/ml in 2021, or perinatally acquired HIV [21]. Third vaccine doses were administered approximately 6.5 months after the second dose.

**Table 1:** Participant characteristics.

Characteristic^a^	PWH (*n* = 64)	Controls (*n* = 117)
HIV-related variables		
Receiving antiretroviral therapy [*n* (%)]	64 (100%)	–
Recent plasma viral load, copies HIV RNA/ml, median (IQR)	<50 (<50–<50)	–
Recent CD4^+^ T-cell count in cells/μl [median (IQR)]	645 (473–958)	–
Nadir CD4^+^ T-cell count in cells/μl [median (IQR)]	225 (95–485)	–
Sociodemographic and health variables		
Age in years [median (IQR)]	57 (42–65)	47 (35–72)
Female sex at birth [*n* (%)]	6 (9.4%)	85 (73%)
White ethnicity [*n* (%)]	46 (72%)	64 (55%)
Number of chronic conditions [median (IQR)]^b^	1 (1–3)	0 (0–1)
Vaccine details		
Initial regimen		
mRNA only [*n* (%)]	67 (81.2%)	114 (97.4%)
ChAdOx1 only [*n* (%)]	6 (9.4%)	1 (0.9%)
ChAdOx1/mRNA [*n* (%)]	6 (9.4%)	2 (1.7%)
Third dose		
BNT162b2 [*n* (%)]	20 (31.2%)	45 (38.5%)
mRNA-1273 [*n* (%)]	44 (68.8%)	72 (61.5%)
Days between second and third doses [median (IQR)]	191 (182–240)	198 (171–218)
Specimen collection		
1 month after third dose [*n* (%)]	63 (98%)	116 (99%)
3 months after third dose [*n* (%)]	58 (91%)	116 (99%)
6 months after third dose [*n* (%)]	55 (86%)	110 (94%)
Post-third dose SARS-CoV-2 infections [*n* (%)]	24 (37.5%)	45 (38.5%)

IQR, interquartile range.

a Sociodemographic, health and vaccine data were collected by self-report and confirmed through medical records wherever available.

b Chronic conditions were defined as hypertension, diabetes, asthma, obesity, chronic diseases of lung, liver, kidney, heart or blood, cancer, and immunosuppression because of chronic conditions or medication.

A total of 24 (38%) PWH and 45 (39%) controls experienced their first SARS-CoV-2 infection between 1 and 6 months post-third dose (one control experienced two infections during this period [22]). Based on the 56 (81%) participants for whom infection timing was available, infections occurred a median 105 (IQR 76–137) days post-third dose, or a median date of 10 April 2022 (IQR 28 February to 04 May), with no temporal differences between PWH and controls (*P* = 0.4). Though SARS-CoV-2 variant information is unavailable for individual infections, most were likely Omicron BA.1 or BA.2 based on local epidemiology at the time [18].

### Longitudinal binding IgG responses following three-dose vaccination

**Fig. 1:**
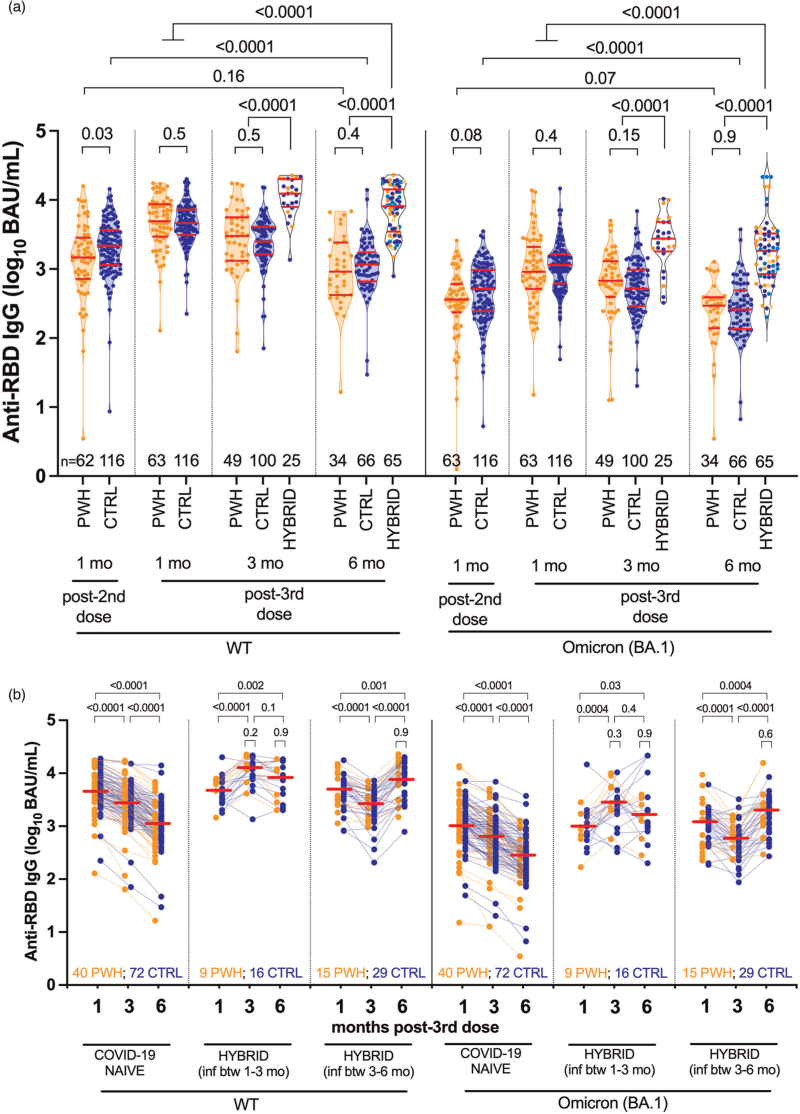
Wild-type-specific and Omicron BA.1-specific anti-RBD IgG concentrations following three-dose coronavirus disease 2019 vaccination.

As reported previously, wild-type-specific serum IgG concentrations were comparable in COVID-19-naive PWH and controls 1 month post-third dose (and also not significantly different post-second dose after adjustment for sociodemographic, health and vaccine-related variables [14,17]) (Fig. 1a). Wild-type-specific IgG concentrations 1 month post-third dose were 3.70 (IQR 3.47–3.94) log_10_ BAU/ml in COVID-19-naive PWH and 3.67 (IQR 3.50–3.86) log_10_ BAU/ml in controls, respectively (*P* = 0.5). Following this, wild-type-specific IgG responses declined comparably in COVID-19-naive PWH and controls. At 3 months, wild-type-specific IgG responses in PWH and controls declined to a median 3.48 (IQR 3.12–3.75) and 3.40 (IQR 3.21–3.61) log_10_ BAU/ml, respectively (*P* = 0.5), while by 6 months, responses had declined to a median 2.96 (IQR 2.62–3.38) and 3.06 (IQR 2.82–3.24) log_10_ BAU/ml in PWH and controls, respectively (*P* = 0.4) (Fig. 1a; also see Fig. 1b). By 6 months post-third dose, wild-type-specific IgG concentrations in COVID-19-naive PWH had declined to levels comparable with those initially elicited by two vaccine doses (*P* = 0.16), whereas those in controls had declined to significantly lower than post-second dose levels (*P* < 0.0001) (Fig. 1a).

Panel a: longitudinal serum anti-RBD IgG concentrations specific to wild-type (left side) and Omicron BA.1 (right side) in COVID-19-naive people with HIV (PWH; orange circles) and controls (CTRL; blue circles). Any participant who experienced a SARS-CoV-2 breakthrough infection between 1 and 3, or 3 and 6 months post-third dose was reclassified into the ‘hybrid’ group at their subsequent study visit, with PWH in orange and CTRL in blue circles. At 6 months post-third dose, the darker colored symbols in the hybrid group denote recent infections that occurred between 3 and 6 months, while the lighter colored symbols denote the infections that had previously occurred between 1 and 3 months. Red bars indicate median and IQR. Comparisons between independent groups were performed using the Mann–Whitney *U* test; longitudinal paired comparisons were performed using the Wilcoxon matched pairs test. *P* values are not corrected for multiple comparisons. Panel b: same data as panel a, but where serum anti-RBD IgG concentrations are plotted longitudinally by participant (PWH in orange, CTRL in blue). Participants are stratified into three groups: those who remained COVID-19-naive throughout the study, those who experienced a SARS-CoV-2 breakthrough infection between 1 and 3 months post-third dose, those who experienced a SARS-CoV-2 breakthrough infection between 3 and 6 months post-third dose. Horizontal red lines denote the overall median response at each time point, where PWH and CTRL are treated as a combined group. *P* values above larger brackets compare responses between time points using the Wilcoxon matched pairs test, again where PWH and CTRL are treated as a combined group. In contrast, the *P* values above the small brackets compare responses between PWH and CTRL with hybrid immunity, at each time point post-SARS-CoV-2 infection, using the Mann–Whitney *U* test. *P* values are not corrected for multiple comparisons.

Similarly, Omicron BA.1-specific IgG responses were comparable in COVID-19-naive PWH and controls at all post-third dose time points (all comparisons *P* ≥ 0.15; Fig. 1a). Nevertheless, BA.1-specific responses were significantly lower than wild-type-specific responses in all groups at all time points (all within-group comparisons of wild-type- and BA.1-specific responses were *P* < 0.0001; not shown). One month post-third dose, for example, BA.1-specific IgG concentrations were 2.96 (IQR 2.71–3.32) log_10_ BAU/ml in PWH, which was 0.74 log_10_ BAU/ml lower than wild-type-specific concentrations at this time. By 6 months, BA.1-specific IgG concentrations had declined to 2.47 (IQR 2.14–2.59) log_10_ BAU/ml in PWH, which was 0.49 log_10_ BAU/ml lower than wild-type-specific concentrations at this time.

We next performed multivariable analyses adjusting for sociodemographic, health and vaccine-related variables to identify variables associated with wild-type-specific and BA.1-specific IgG concentrations at 6 months post-third dose in the COVID-19-naive subgroup. These analyses revealed that a higher number of chronic health conditions – but not HIV infection – was associated with poorer IgG responses at this time (*P* = 0.028 for wild-type-specific responses; 0.016 for BA.1-specific responses), as was male sex (*P* = 0.012 for BA.1-specific responses) (Supplementary Table 1). In fact, adjusted IgG concentrations were slightly *higher* in PWH compared with controls, though this was not statistically significant. Of note, receipt of an mRNA-1273 third dose (rather than BNT162b2) was associated with stronger wild-type-specific IgG responses (*P* = 0.029), though not BA.1-specific responses (*P* = 0.13), 6 months post-third dose. Among PWH, we also observed no significant relationship between most recent or nadir CD4^+^ T-cell count and either wild-type-specific or BA.1-specific IgG concentrations at this time (*P* ≥ 0.3; Supplementary Figure 1).

We estimated the half-lives of wild-type-specific IgG following three-dose vaccination to be a median 66 (IQR 47–89) days in COVID-19-naive PWH compared to 72 (IQR 54–96) days in controls, a difference that was not statistically significant (*P* = 0.2; Supplementary Figure 2). Estimated BA.1-specific IgG half-lives were also comparable, at a median 71 (IQR 49–104) days for PWH compared with 74 (IQR 59–90) days in controls (*P* = 0.8). Multivariable analyses confirmed that HIV infection was not associated with wild-type-specific or BA.1-specific IgG half-lives post-third dose (Supplementary Table 2). Among COVID-19-naive PWH, we initially observed a weak inverse correlation between recent CD4^+^ T-cell count and IgG half-life, but this was not significant after excluding an outlier with a long (>200 day) half-life (Supplementary Figure 1).

By contrast, the nearly 40% of PWH and controls who experienced their first SARS-CoV-2 infection between 1 and 6 months post-third dose exhibited markedly higher wild-type-specific and BA.1-specific IgG concentrations than their COVID-19-naive counterparts at all post-infection time points (all comparisons *P* < 0.0001 in Fig. 1a; also see Fig. 1b). In fact, at 6 months post-third dose, IgG responses in this ‘hybrid immunity’ group were significantly higher than those initially induced by vaccination alone, for example, BA.1-specific IgG concentrations were a median 3.26 log_10_ BAU/ml (IQR 2.93–3.52), which was 0.27 log_10_ BAU/ml higher than at 1 month post-third dose (*P* < 0.0001). Importantly, the magnitude of these ‘hybrid’ IgG responses was comparable between PWH and controls at all post-infection time points tested (all *P* ≥ 0.2; Fig. 1b, see *P* values above small brackets). Notably, while most participants experienced a marked boost in antibody levels following SARS-CoV-2 infection, IgG responses in a minority of PWH and controls remained constant or even declined post-infection (Fig. 1b).

### Longitudinal ACE2 displacement activity following three-dose vaccination

**Fig. 2:**
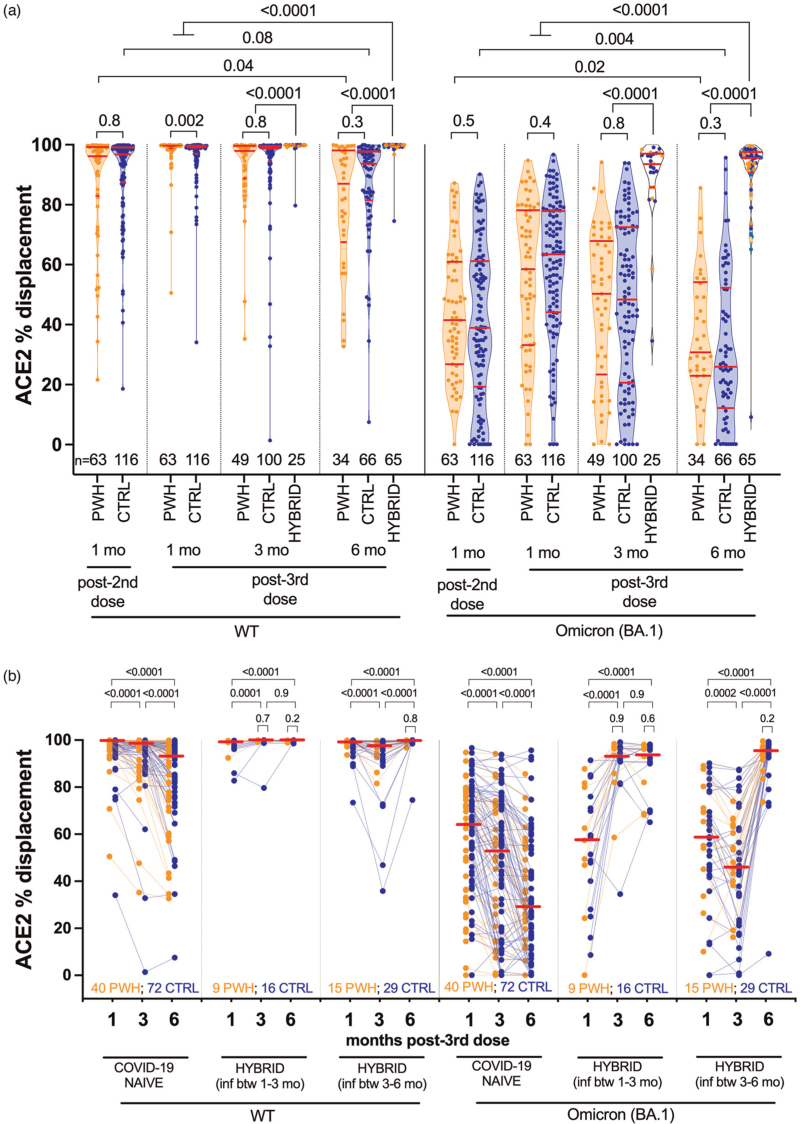
Wild-type-specific and Omicron BA.1-specific ACE2 displacement function following three-dose COVID-19 vaccination.

One month post-third dose, wild-type-specific ACE2 displacement activity in COVID-19-naive PWH was a median 99.6% (IQR 98.7–99.8%) compared with 99.1% (IQR 97–99.6%) in controls (univariable *P* = 0.002; Fig. 2a), though this did not remain significant after multivariable adjustment (not shown). Following this, wild-type-specific ACE2 displacement activities declined similarly in both COVID-19-naive groups: at three months, activities had decreased to a median 97.9% (IQR 88.8–99.6) in PWH versus 98.8% (IQR 94.5–99.5) in controls (*P* = 0.8), while by 6 months, activities had decreased to a median 87% (IQR 67.5–98.1) in PWH versus 93.6% (IQR 81.5–97.8) in controls (*P* = 0.3), levels that were comparable or lower than after two-dose vaccination (all *P* < 0.1) (Figs. 2a and b).

Panel a: same as Fig. 1a, but for ACE2 displacement activity in serum, a surrogate measure of virus neutralization, where results are reported in terms of % ACE2 displacement. Panel b: same data as panel a, but where ACE2% displacement activities are plotted longitudinally by participant (PWH in orange, CTRL in blue). The legend is the same as for Fig. 1b.

BA.1-specific responses remained significantly lower than wild-type-specific responses at all time points (all within-group comparisons for wild-type-specific and BA.1-specific responses *P* < 0.0001; not shown), but these responses also declined similarly in COVID-19-naive PWH and controls (Fig. 2a). Between 1 and 6 months post-third dose, for example, BA.1-specific ACE2 displacement activities decreased from a median 58.5% (IQR 33.2–78.2%) in PWH and 63.4% (IQR 44.1–77.9%) in controls (*P* = 0.4), to 30.8% (IQR 23.0–54.1) in PWH and 25.9% (IQR 12.1–52.2) in controls (*P* = 0.3), where the latter values were significantly lower than those observed after two-dose vaccination (all comparisons *P* ≤ 0.02).

Multivariable analyses confirmed that, among COVID-19-naive participants, HIV infection was not associated with either wild-type-specific or BA-1-specific ACE2 displacement activities at 6 months post-third dose (Supplementary Table 3). Rather, having received a mRNA-1273 third dose was the only independent correlate of stronger wild-type-specific ACE2 displacement activity at this time (*P* = 0.0025). There was also no evidence that a low recent or nadir CD4^+^ T-cell count was associated with lower wild-type-specific or BA.1-specific ACE2 displacement activities 6 months post-third dose in COVID-19-naïve PWH (in fact, we observed a weak inverse relationship between nadir CD4^+^ T-cell count and Omicron BA.1-specific ACE2 displacement at this time; Supplementary Figure 1). This was consistent with prior observations at 1 month post-third dose, which we attributed to the observation that PWH with low nadir CD4^+^ T-cell counts were eligible for the higher (100 μg) third mRNA-1273 dose [14].

Similar to IgG concentrations, a SARS-CoV-2 breakthrough infection markedly boosted wild-type-specific and BA.1-specific ACE2 displacement activities at both 3 and 6 months post-third dose (all *P* < 0.0001), where activities at 6 months in this group were overall significantly greater than peak responses induced by three-dose vaccination (both *P* < 0.0001) (Fig. 2a). For instance, BA.1-specific ACE2 displacement activity was 95.5% (IQR 90.6–97.6) in the hybrid group 6 months post-third dose, which was 31% higher than that elicited by vaccination alone. Importantly, the magnitude of these ‘hybrid’ ACE2 displacement responses was comparable between PWH and controls at all post-infection time points tested (all *P* ≥ 0.2; Fig. 2b, see *P* values above small brackets**)**. It is again notable, however, that a minority of PWH and controls did not show appreciable increases in this activity following infection (Fig. 2b).

### Longitudinal viral neutralization activity following three-dose vaccination

**Fig. 3:**
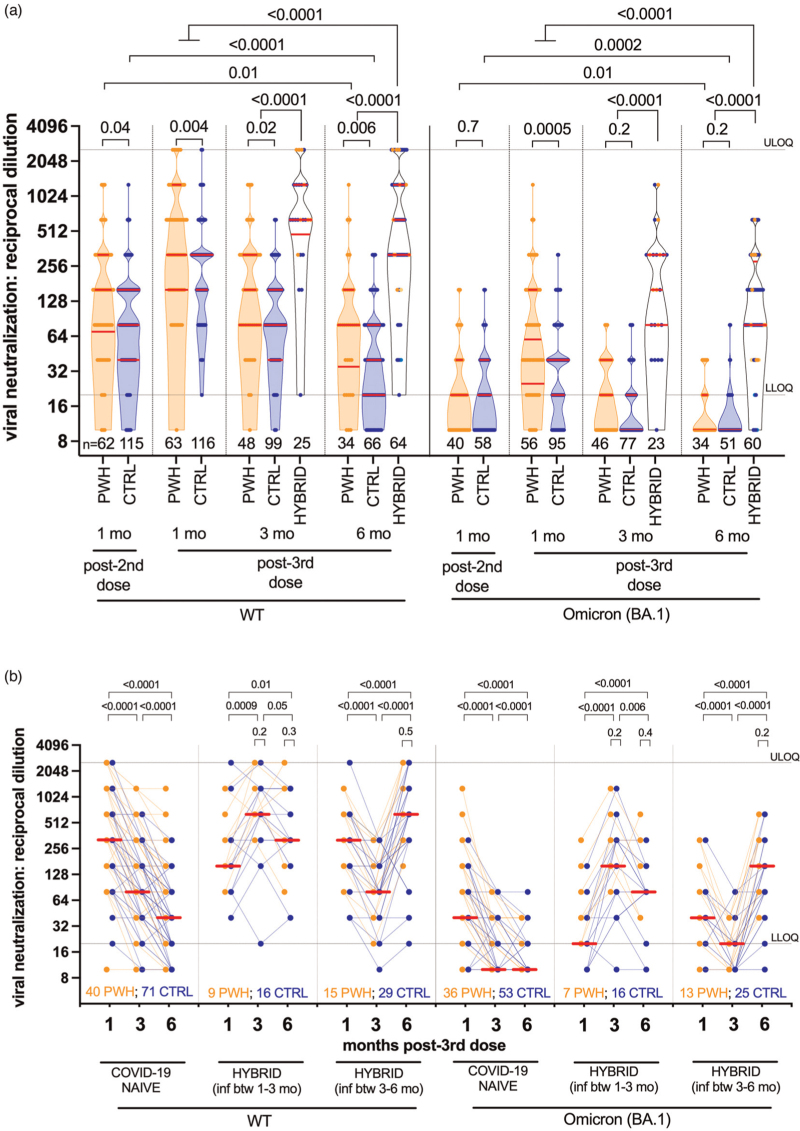
Wild-type-specific and Omicron BA.1-specific live virus neutralization activity following three-dose coronavirus disease 2019 vaccination.

One month post-third dose, wild-type-specific neutralization activity in PWH (median reciprocal plasma dilution 320; IQR 160–1280) was slightly higher than in controls (median 320, IQR 160–320; *P* = 0.004) (Fig. 3a), though this did not remain significant after multivariable adjustment as previously reported [14]. COVID-19-naive PWH continued to maintain higher wild-type-specific neutralization throughout follow-up: by 3 months, neutralization declined to 160 (IQR 80–320) in PWH compared with 80 (IQR 40–160) in controls (*P* = 0.02), while by 6 months, neutralization had declined to a median 80 (IQR 35–160) in PWH and 40 (IQR 20–80) in controls (*P* = 0.006), values that were below the levels originally elicited by two-dose vaccination (both groups *P* ≤ 0.01). As reported previously [14], we attribute the higher (univariable) post-third dose neutralization in PWH to the fact that the majority of PWH met one or more of the eligibility criteria for receipt of a full (100 μg) third dose of mRNA-1273, rather than the standard 50 μg mRNA-1273 booster that was offered to the general population. Indeed, after adjustment for sociodemographic, health and vaccine-related variables (including third-dose vaccine brand), HIV infection did not remain significantly associated with wild-type-specific neutralization activity at 6 months post-third dose (Supplementary Table 4). Rather, a higher number of health conditions was the only variable independently associated with poorer wild-type-specific neutralization at this time.

Panel a: same as Fig. 1a, but for live virus neutralization activity, defined as the highest reciprocal plasma dilution at which neutralization was observed in all wells of a triplicate assay. Serial two-fold dilutions of 1/20 (lower limit of quantification; LLOQ) to 1/2560 (upper limit of quantification; ULOQ) were tested. Plasma samples showing neutralization in fewer than three wells at a 1/20 dilution are displayed as a reciprocal dilution of ‘10’ and were reported as below limit of quantification (BLOQ) in the text. Omicron BA.1-specific neutralization was performed on a subset of samples only. Panel b: same data as panel a, but where neutralization activities are plotted longitudinally by participant (PWH in orange, CTRL in blue). The legend is the same as for Fig. 1b. Note that many data points are superimposed in both panels.

BA.1-specific neutralization was significantly lower than wild-type responses at all timepoints for all groups (all *P* < 0.0001; not shown), though responses in PWH were not further impaired compared with controls. In fact, 1 month post-third dose, BA.1-specific neutralization was higher in PWH compared with controls: a median 60 (IQR 25–160) and 40 (IQR 20–40), respectively (*P* = 0.0005), though these values were five-fold to eight-fold lower than corresponding wild-type-specific responses. BA.1-specific neutralization subsequently declined rapidly; at 3 months, this was BLOQ in 67.4% of COVID-19-naive PWH and 80.5% of controls (*P* = 0.2), whereas at 6 months, this was BLOQ in 82.4% of PWH and 92.2% of controls (*P* = 0.2), levels that were significantly lower than peak responses after two-dose vaccination (all comparisons *P* ≤ 0.01). In multivariable analyses, older age – but not HIV infection – was the only significant correlate of poorer BA.1-specific neutralization among COVID-19-naive individuals at 6-month post-third dose (*P* = 0.045; Supplementary Table 4). We also observed no significant associations between CD4^+^ T-cell parameters and either wild-type-specific or BA-1-specific neutralization at 6 months among COVID-19-naive PWH (*P* ≥ 0.3; Supplementary Figure 1).

By contrast, individuals who experienced breakthrough infection showed significantly stronger wild-type-specific and BA.1-specific neutralization compared with their COVID-19-naive counterparts at 3 and 6 months (all *P* < 0.0001; Fig. 3a), responses that were significantly higher than those induced by vaccination alone (all comparisons *P* ≤ 0.0001) (Fig. 3a). At 6 months, for example, BA.1-specific responses in participants with hybrid immunity were a median 80 (IQR 80–280), compared with the cohort median of 40 (IQR 20–80) 1 month post-third dose. Importantly, the magnitude of these ‘hybrid’ neutralization responses was comparable between PWH and controls at all post-infection time points tested (all *P* ≥ 0.2; Fig. 3b, see *P* values above small brackets).

### Responses to newer Omicron variants: BA.5

**Fig. 4:**
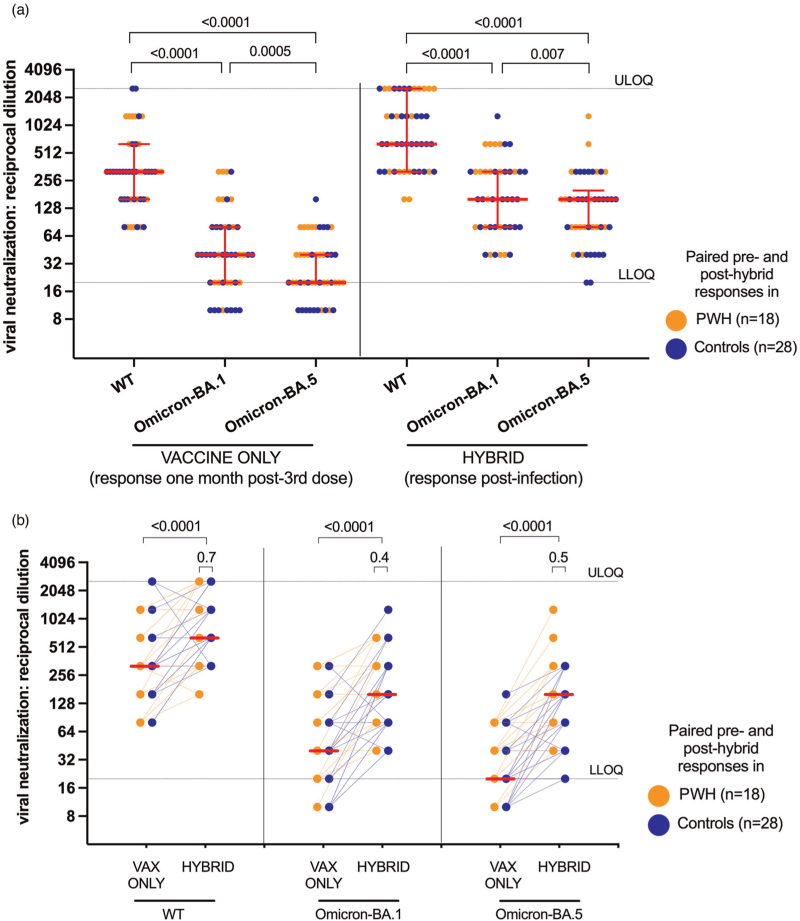
Wild-type-specific, Omicron BA.1-specific and Omicron BA.5-specific live virus neutralization activity before and after breakthrough infection.

Since the emergence of Omicron BA.1, even more immune evasive variants have developed, including Omicron BA.5 that has dominated globally [23–26]. We, therefore, longitudinally assessed BA.5-specific neutralization activity in a subset of 18 PWH and 28 controls who experienced breakthrough infections, that were likely caused by BA.1 or BA.2 based on local epidemiology [18]. One month post-third dose, and prior to SARS-CoV-2 infection, the median reciprocal dilution required for BA.5 neutralization was 20 (IQR BLOQ-20) in this subset, which was two-fold lower than that for BA.1 (median 40, IQR 20–80; *P* = 0.0005), and 16-fold lower than that for wild-type (median 320, IQR 160–640; *P* < 0.0001) (Fig. 4a). Although neutralization activity against all three virus strains rose significantly post-infection, (all *P* < 0.0001; Fig. 4b), BA.5-specific neutralization (median 160, IQR 80–200) remained lower than that against BA.1 (median 160, IQR 80–320; *P* = 0.007) and wild-type (median 640, IQR 320–2560; *P* < 0.0001) (Fig. 4a). Importantly, no significant differences were observed between PWH and controls in their ability to neutralize any virus strains after post-vaccination SARS-CoV-2 infection (all *P* ≥ 0.4) (Fig. 4b, see *P* values above small brackets).

Panel a: wild-type-specific, Omicron BA.1-specific and Omicron BA.5-specific neutralization activities in 18 PWH (orange circles) and 28 CTRL (blue circles) 1 month after the third vaccine dose (’vaccine only’) and after subsequent SARS-CoV-2 infection (’hybrid’). In this panel, data are plotted to facilitate comparisons between variant-specific neutralization activities, before and after breakthrough infection. *P* values, computed on PWH and CTRL as a combined group, were computed using the Wilcoxon matched pairs test. *P* values are not corrected for multiple comparisons. ULOQ/LLOQ: upper/lower limit of quantification. Panel b: same data as in panel a but plotted longitudinally by participant to highlight the impact of breakthrough infection on neutralization activity. Red lines indicate medians of PWH and CTRL as a combined group. *P* values on top of large brackets compare paired responses before and after breakthrough infection using the Wilcoxon matched pairs test. In contrast, the *P* values above the small brackets compare responses *between* PWH and CTRL at the post-infection time point using the Mann–Whitney *U* test. Note that a large number of points are superimposed in this plot.

## Discussion

Our results demonstrate that antibody response durability following three-dose COVID-19 vaccination in COVID-19-naive PWH receiving suppressive ART is comparable to controls without HIV. As we reported previously [14,17], initial peak antibody responses after three-dose vaccination were similar between PWH and controls, and our new data additionally show that wild-type-specific and Omicron-specific IgG concentrations, ACE2 displacement and virus neutralization activities declined at similar rates among PWH and controls who remained COVID-19-naive throughout follow-up. Multivariable analyses adjusting for sociodemographic, health and vaccine-related variables confirmed that there was no significant impact of HIV infection on any antibody outcome measure. Nevertheless, by 6 months post-third dose, all antibody responses in COVID-19-naive participants, regardless of HIV status, had declined to similar or lower levels than peak responses observed after two vaccine doses. In fact, as early as 3 months post-third dose, BA.1-specific neutralization was already below the limit of quantification in ∼75% of COVID-19-naive participants, regardless of HIV status. Moreover, and consistent with recent reports [27–29], the ability to neutralize BA.5 was even poorer than BA.1.

By contrast, almost all participants who experienced their first SARS-CoV-2 infection (presumably BA.1 or BA.2 [18]) post-third dose displayed enhanced antibody responses to all viral variants tested. In fact, at 6 months post-third dose, all antibody measures in breakthrough infection cases were on average higher than those elicited by vaccination alone. Importantly, the magnitude of these ‘hybrid’ responses was equally strong in PWH and controls. Nevertheless, it was notable that responses to BA.5 remained significantly lower than those against wild-type and BA.1 even after infection, and that a minority of participants failed to show improved antibody responses post-infection, a phenomenon which requires further study.

Our study has several limitations. Our observations may not be generalizable to PWH with low CD4^+^ T-cell counts and/or who are not receiving suppressive ART. Indeed, results from several studies indicate that PWH with CD4^+^ T-cell counts below 500 cells/μl mount weaker responses to the first [30–32] and second [31–38] doses of COVID-19 vaccine. We did not assess cellular immunity in participants. We were unable to compare the duration or severity of breakthrough SARS-CoV-2 infections among PWH and controls as we did not systematically collect this information. Given recent reports that post-third dose breakthrough infections may be more frequent in PWH [39], future studies should address this. Finally, any recommendations based on our results are limited by the ongoing emergence of new SARS-CoV-2 variants, including strains that may be more immune-evasive than BA.5 (e.g. BA.2.75) [40–44].

In conclusion, our observations confirm the humoral immune benefits of third COVID-19 vaccine doses in PWH receiving suppressive ART, and further reveal that the durability of third-dose responses is comparable to that in persons without HIV. Nevertheless, regardless of HIV status, individuals who remain COVID-19-naive will benefit from a fourth dose within 3–6 months of their third dose, as antibody concentrations and neutralization activities declined markedly over time in this group, and the ability of vaccine-induced responses to neutralize the dominant Omicron BA.5 variant were even poorer than BA.1. By contrast, the majority of individuals who experienced their first SARS-CoV-2 infection post-third vaccine dose showed significantly higher antibody activities than those induced by vaccination alone (though anti-BA.5 responses remained weaker than anti-BA.1 responses, even after breakthrough infection). These observations suggest that a slightly delayed fourth dose (e.g. to 3–6 months following infection) would optimally benefit this group. Further studies of hybrid response durability are required, as are direct comparisons with immune responses elicited by a fourth dose in COVID-19-naive individuals, particularly in light of new bivalent formulations that include wild-type and Omicron Spike antigens.

## Acknowledgements

This work is dedicated to the memory of our friend and colleague Hesham Ali who sadly passed away in July 2022. We thank the phlebotomists and laboratory staff at the BC Centre for Excellence in HIV/AIDS, the Hope to Health Research and Innovation Centre, St. Paul's Hospital, and Simon Fraser University for assistance. Above all, we thank the participants, without whom this study would not have been possible.

Author contributions: M.A.B. and Z.L.B. are co-principal investigators and conceived the study, with C.T.C., C.C., A.H.A., V.L., M.H., M.G.R., R.G., S.G., J.S.G.M., M.H. and M.H. additionally contributing to study design and development. M.A.B., Z.L.B., M.G.R., A.H.A., C.T.C. and C.C. obtained project funding. M.A.B., Z.L.B., H.R.L., F.M. and P.K.C. designed experiments. H.R.L., R.M., P.K.C., Y.S., F.Y., S.S., E.B., N.M.-G., S.D., M.C.D., R.K., S.E., L.Y., B.G., F.H.O., G.U., J.T., P.S., L.B. and C.J.B. contributed to specimen collection, data collection, data curation and/or data analysis. H.R.L., Y.S., D.H., M.L.D., J.S., M.N., M.G.R., M.A.B. and Z.L.B. supervised the research, laboratory assays and/or contributed to project or cohort management. Z.L.B. performed statistical analyses with support from C.J.B.. N.P., C.F.L., M.G.R., W.D., C.J.B., and M.N. provided, generated and/or validated local Omicron isolates. H.R.L. wrote the first draft of the manuscript.

Funding: this work was supported by funding from Genome BC, Michael Smith Health Research BC, and the BCCDC Foundation for Public Health through a rapid SARS-CoV-2 vaccine research initiative in BC award (VAC-009 to Z.L.B., M.A.B.). It was also supported by the Public Health Agency of Canada (PHAC) through two COVID-19 Immunology Task Force (CITF) COVID-19 Awards (the first to Z.L.B., M.G.R., M.A.B. and the second to C.T.C., C.C., A.H.A.). Additional funding was received from the Canadian Institutes for Health Research (GA2-177713; to M.A.B.), the Coronavirus Variants Rapid Response Network (FRN-175622; to M.A.B.), the Canada Foundation for Innovation through two Exceptional Opportunities Fund COVID-19 awards (the first to C.J.B., C.F.L., M.L.D., and the second to M.N., M.A.B., Z.L.B.), a British Columbia Ministry of Health–Providence Healthcare Research Institute COVID-19 Research Priorities Grant (to C.J.B. and C.F.L.) and the CIHR Canadian HIV Trials Network (CTN) (to A.H.A.). F.M. is supported by a fellowship from the CIHR Canadian HIV Trials Network. M.L.D. and Z.L.B. hold Scholar Awards from Michael Smith Health Research BC. F.Y. and E.B. were supported by SFU Undergraduate Research Awards. M.D. is supported by a CIHR Canada Graduate Scholarships-Master's award. G.U. and F.H.O. are supported by PhD fellowships from the sub-Saharan African Network for TB/HIV Research Excellence (SANTHE), a DELTAS Africa Initiative (grant # DEL-15-006). The DELTAS Africa Initiative is an independent funding scheme of the African Academy of Sciences (AAS)'s Alliance for Accelerating Excellence in Science in Africa (AESA) and supported by the New Partnership for Africa's Development Planning and Coordinating Agency (NEPAD Agency) with funding from the Wellcome Trust (grant # 107752/Z/15/Z) and the UK government. The views expressed in this publication are those of the authors and not necessarily those of PHAC, CITF, AAS, NEPAD Agency, Wellcome Trust, the Canadian or UK governments or other funders.

Posted history: this manuscript is posted to MedRxiv: https://doi.org/10.1101/2022.11.03.22281912

### Conflicts of interest

There are no conflicts of interest.

## Supplementary Material

Supplemental Digital Content

## Supplementary Material

Supplemental Digital Content

## Supplementary Material

Supplemental Digital Content

## Supplementary Material

Supplemental Digital Content

## Supplementary Material

Supplemental Digital Content

## Supplementary Material

Supplemental Digital Content
