# Supplementary material for: Antibody response durability following three-dose coronavirus disease 2019 vaccination in people with HIV receiving suppressive antiretroviral therapy
Source: AIDS. 2022 Dec 22;37(5):709–21. doi: 10.1097/QAD.0000000000003469 (PMC9994797; doi:10.1097/QAD.0000000000003469)
Supplement: Supplemental Digital Content [file aids-37-709-s001.pdf]

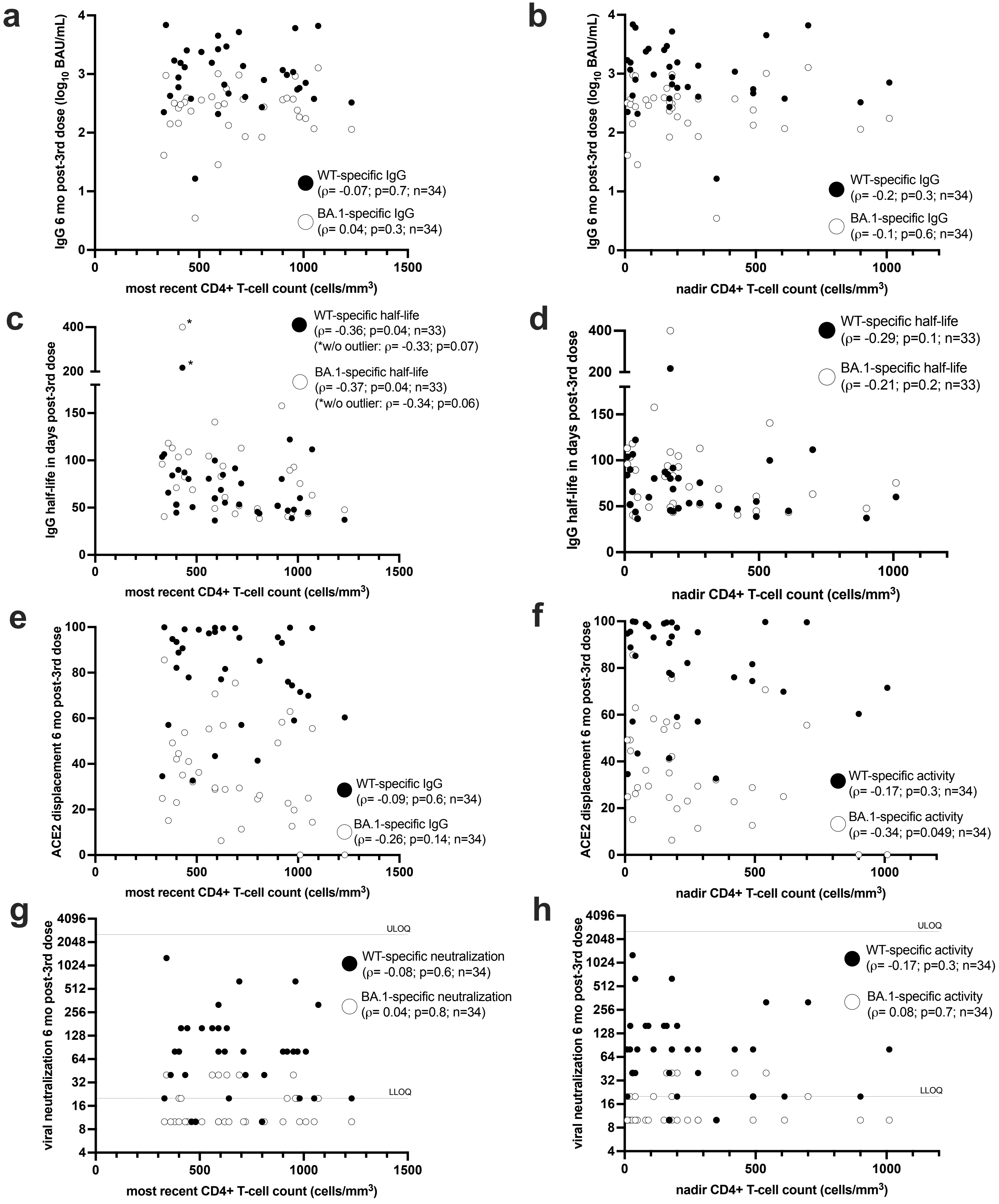

Supplementary Figure 1: Relationships between most recent and nadir CD4+ T-cell counts and humoral responses six months post-third vaccine dose. Relationships were assessed using Spearman's correlation. Measurements against wild-type (WT) SARS-CoV-2 are indicated by black symbols; measurements against Omicron BA.1 are indicated by open symbols. Analyses are restricted to COVID-19-naïve PWH. ULOQ/LLOQ: upper/lower limit of quantification
