# Supplementary material for: Antibody response durability following three-dose coronavirus disease 2019 vaccination in people with HIV receiving suppressive antiretroviral therapy
Source: AIDS. 2022 Dec 22;37(5):709–21. doi: 10.1097/QAD.0000000000003469 (PMC9994797; doi:10.1097/QAD.0000000000003469)
Supplement: Supplemental Digital Content [file aids-37-709-s002.pdf]

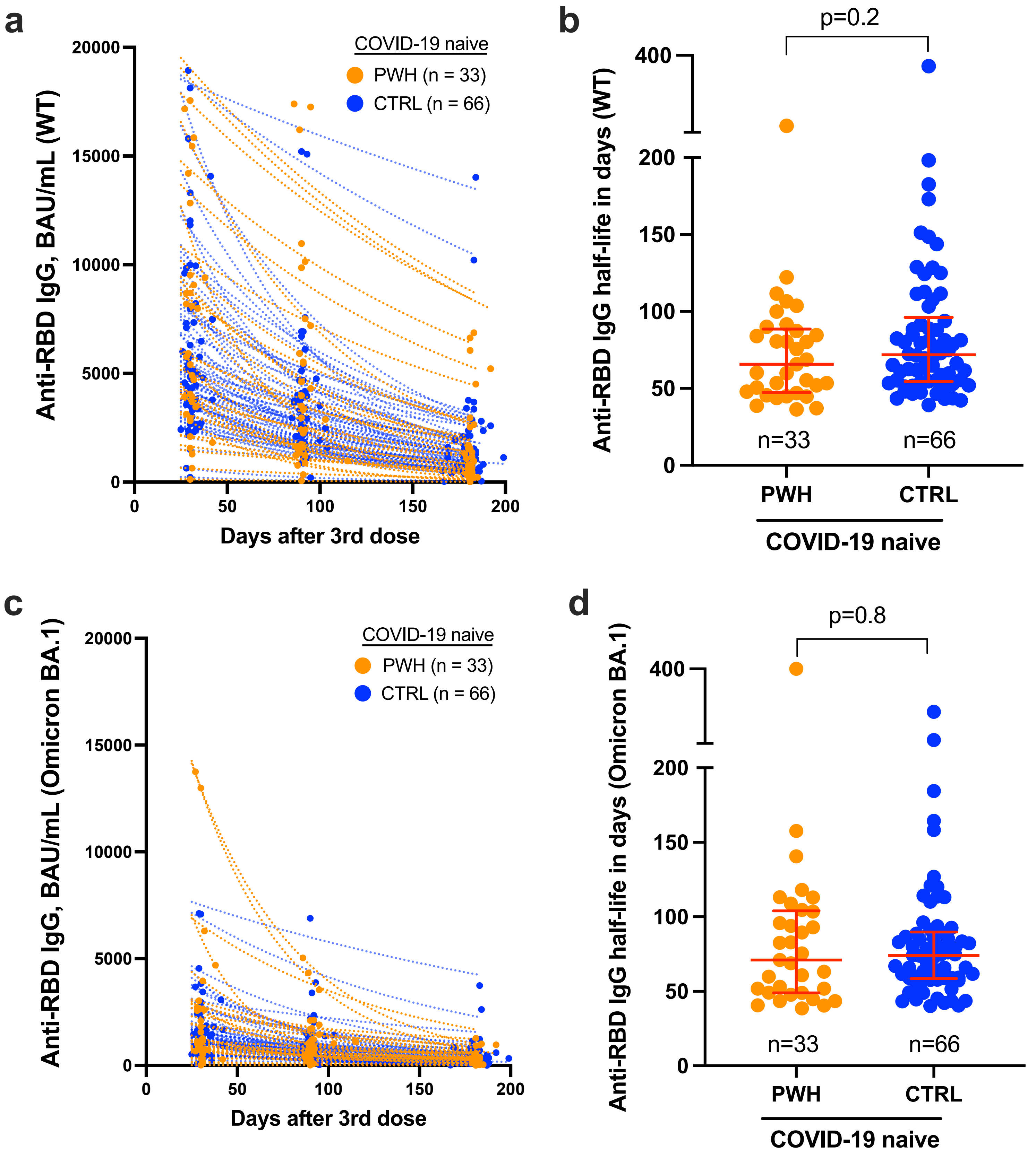

Supplementary Figure 2: Temporal declines in WT- and Omicron BA.1-specific anti-RBD IgG concentrations following three-dose COVID-19 vaccination. *Panel A:* Temporal declines in WT-specific anti-RBD IgG concentrations following three-dose vaccination in PWH (orange) and CTRL (blue) who remained COVID-19-naïve during follow-up, and who completed all post-third dose visits. *Panel B:* Estimated WT-specific IgG half-lives following three-dose vaccination, calculated by fitting an exponential curve to each participant's data in panel B. Red bars and whiskers represent the median and IQR. P-values were computed using the Mann-Whitney U-test. *Panel C:* Same as panel A, but for Omicron (BA.1)-specific anti-RBD IgG concentration. *Panel D:* Same as panel B, but for half-lives calculated from the BA.1-specific data.
