# Supplementary material for: Antibody response durability following three-dose coronavirus disease 2019 vaccination in people with HIV receiving suppressive antiretroviral therapy
Source: AIDS. 2022 Dec 22;37(5):709–21. doi: 10.1097/QAD.0000000000003469 (PMC9994797; doi:10.1097/QAD.0000000000003469)
Supplement: Supplemental Digital Content [file aids-37-709-s003.docx]

**Supplementary Table 1: Multivariable linear regression analysis of the relationship between sociodemographic, health and vaccine-related variables on WT- and BA.1-specific IgG concentrations six months post-third COVID-19 vaccine dose.**

| **Immunogenicity outcome**^a^ | **Variable** | **SARS-CoV-2 Variant** | | | | | |
| --- | --- | --- | --- | --- | --- | --- | --- |
|  |  | **Wild-type** | | | **Omicron BA.1** | | |
|  |  | **Estimate** | **95% CI** | **p-value** | **Estimate** | **95% CI** | **p-value** |
| **Anti-RBD IgG (log_10_BAU/ml)** | HIV infection | 0.14 | -0.12 to 0.39 | 0.29 | 0.23 | -0.019 to 0.49 | 0.069 |
| **6 months post-third dose** | Age (per year) | 0.0021 | -0.0042 to 0.0084 | 0.5 | 0.0018 | -0.0044 to 0.0081 | 0.56 |
|  | Male sex | -0.21 | -0.43 to 0.0083 | 0.059 | -0.28 | -0.5 to -0.064 | **0.012** |
|  | White ethnicity | -0.092 | -0.29 to 0.1 | 0.35 | 0.0022 | -0.19 to 0.2 | 0.98 |
|  | # Chronic conditions (per additional) | -0.14 | -0.26 to -0.015 | **0.028** | -0.15 | -0.27 to -0.029 | **0.016** |
|  | Dual ChAdOx1 as initial regimen | -0.089 | -0.42 to 0.24 | 0.59 | -0.16 | -0.49 to 0.17 | 0.33 |
|  | mRNA-1273 as 3rd dose | 0.22 | 0.023 to 0.41 | **0.029** | 0.15 | -0.044 to 0.34 | 0.13 |
|  | Interval btw 2nd and 3rd doses (per day) | 0.0011 | -0.0018 to 0.004 | 0.44 | 0.0015 | -0.0014 to 0.0044 | 0.3 |

^a^Analysis was restricted to participants who remained COVID-19 naïve six months post-third vaccine dose
