# Supplementary material for: Antibody response durability following three-dose coronavirus disease 2019 vaccination in people with HIV receiving suppressive antiretroviral therapy
Source: AIDS. 2022 Dec 22;37(5):709–21. doi: 10.1097/QAD.0000000000003469 (PMC9994797; doi:10.1097/QAD.0000000000003469)
Supplement: Supplemental Digital Content [file aids-37-709-s004.docx]

**Supplementary Table 2: Multivariable linear regression analysis of the relationship between sociodemographic, health and vaccine-related variables on WT- and BA.1-specific IgG half-lives following three-dose COVID-19 vaccination.**

| **Immunogenicity outcome**^a^ | **Variable** | **SARS-CoV-2 Variant** | | | | | |
| --- | --- | --- | --- | --- | --- | --- | --- |
|  |  | **Wild-type** | | | **Omicron BA.1** | | |
|  |  | **Estimate** | **95% CI** | **p-value** | **Estimate** | **95% CI** | **p-value** |
| **Anti-RBD IgG half-lives** | HIV infection | 1.7 | -25 to 28 | 0.9 | 12 | -18 to 42 | 0.42 |
| **post-3rd dose, in days** | Age (per year) | 0.41 | -0.26 to 1.1 | 0.23 | 0.33 | -0.42 to 1.1 | 0.38 |
|  | Male sex | -21 | -44 to 2.1 | 0.075 | -12 | -39 to 14 | 0.35 |
|  | White ethnicity | 3.9 | -17 to 25 | 0.71 | 6.5 | -17 to 30 | 0.58 |
|  | # Chronic conditions (per additional) | -8.3 | -21 to 4.3 | 0.19 | -9.1 | -23 to 5.1 | 0.21 |
|  | Dual ChAdOx1 as initial regimen | -0.31 | -35 to 35 | 0.99 | -2.7 | -42 to 37 | 0.89 |
|  | mRNA-1273 as 3rd dose | 14 | -6.7 to 34 | 0.18 | -3.9 | -27 to 19 | 0.74 |
|  | Interval btw 2nd and 3rd doses (per day) | 0.011 | -0.3 to 0.32 | 0.94 | -0.066 | -0.41 to 0.28 | 0.7 |

^a^Analysis was restricted to participants who remained COVID-19 naïve throughout follow-up, and who completed all three post-third dose study visits
