# Supplementary material for: Antibody response durability following three-dose coronavirus disease 2019 vaccination in people with HIV receiving suppressive antiretroviral therapy
Source: AIDS. 2022 Dec 22;37(5):709–21. doi: 10.1097/QAD.0000000000003469 (PMC9994797; doi:10.1097/QAD.0000000000003469)
Supplement: Supplemental Digital Content [file aids-37-709-s005.docx]

**Supplementary Table 3: Multivariable linear regression analysis of the relationship between sociodemographic, health and vaccine-related variables on WT- and BA.1-specific ACE2 displacement activity six months post-third COVID-19 vaccine dose.**

| **Immunogenicity outcome** | **Variable** | **SARS-CoV-2 Variant** | | | | | |
| --- | --- | --- | --- | --- | --- | --- | --- |
|  |  | **Wild-type** | | | **Omicron BA.1** | | |
|  |  | **Estimate** | **95% CI** | **p-value** | **Estimate** | **95% CI** | **p-value** |
| **ACE2 Displacement (%)** | HIV infection | -1.2 | -11 to 9.1 | 0.82 | 11 | -2.6 to 25 | 0.11 |
|  | Age (per year) | -0.068 | -0.32 to 0.19 | 0.6 | -0.23 | -0.57 to 0.11 | 0.19 |
|  | Male sex | -4.3 | -13 to 4.7 | 0.34 | -11 | -23 to 1 | 0.072 |
|  | White ethnicity | 0.68 | -7.2 to 8.6 | 0.86 | -1.1 | -12 to 9.4 | 0.84 |
|  | # Chronic conditions (per additional) | -4.2 | -9 to 0.73 | 0.094 | -4.2 | -11 to 2.3 | 0.2 |
|  | Dual ChAdOx1 as initial regimen | -3.5 | -17 to 10 | 0.61 | 0.53 | -17 to 18 | 0.95 |
|  | mRNA-1273 as 3rd dose | 12 | 4.5 to 20 | **0.0025** | 5 | -5.6 to 16 | 0.35 |
|  | Interval btw 2nd and 3rd doses (per day) | 0.056 | -0.061 to 0.17 | 0.35 | -0.0051 | -0.16 to 0.15 | 0.95 |

^a^Analysis was restricted to participants who remained COVID-19 naïve six months post-third vaccine dose
