# Supplementary material for: Antibody response durability following three-dose coronavirus disease 2019 vaccination in people with HIV receiving suppressive antiretroviral therapy
Source: AIDS. 2022 Dec 22;37(5):709–21. doi: 10.1097/QAD.0000000000003469 (PMC9994797; doi:10.1097/QAD.0000000000003469)
Supplement: Supplemental Digital Content [file aids-37-709-s006.docx]

**Supplementary Table 4: Multivariable analyses of the relationship between sociodemographic, health and vaccine-related variables on viral neutralization six months following the third vaccine dose.**

| **Immunogenicity outcome^a^** | **Variable** | **SARS-CoV-2 Variant** | | | | | |
| --- | --- | --- | --- | --- | --- | --- | --- |
|  |  | **Wild-type^c^** | | | **Omicron BA.1^c^** | | |
|  |  | **Estimate ^c^** | **95% CI ^c^** | **p-value** | **Odds Ratio ^c^** | **95% CI^c^** | **p-value** |
| **Log_2_ viral neutralization^b^** | HIV infection | 0.96 | -0.36 to 2.3 | 0.15 | 4.9 | 0.67 to 51 | 0.14 |
|  | Age (per year) | -0.0014 | -0.026 to 0.023 | 0.91 | 0.96 | 0.91 to 1 | **0.045** |
|  | Male sex | -0.52 | -1.3 to 0.27 | 0.2 | 0.28 | 0.052 to 1.2 | 0.1 |
|  | White ethnicity | -0.54 | -1.2 to 0.14 | 0.12 | 1.3 | 0.39 to 4.3 | 0.69 |
|  | # Chronic conditions (per additional) | -0.46 | -0.88 to -0.047 | **0.029** | 0.77 | 0.31 to 1.8 | 0.55 |
|  | Dual ChAdOx1 as initial regimen | 0.72 | -0.46 to 1.9 | 0.23 | 3 | 0.52 to 18 | 0.21 |
|  | mRNA-1273 as 3rd dose | 0.36 | -0.32 to 1 | 0.29 | 0.93 | 0.28 to 3.3 | 0.91 |
|  | Interval btw 2nd and 3rd doses (per day) | -0.0019 | -0.012 to 0.0084 | 0.71 | 0.99 | 0.98 to 1 | 0.55 |
|  | EDTA as anticoagulant^d^ | 0.27 | -1.2 to 1.7 | 0.71 | 0.7 | 0.057 to 7.1 | 0.77 |

^a^Analysis was restricted to participants who remained COVID-19 naïve six months post-third vaccine dose

^b^reciprocal plasma dilutions were log_2_ transformed prior to multivariable analysis.

^c^Multivariable analysis for wild-type neutralization was performed using linear regression. Multivariable analysis for Omicron BA.1 neutralization was performed using logistic regression due to a high proportion of measurements below the limit of quantification (BLOQ) at this time point.

^d^Neutralization assays were performed using plasma (not serum as in other analyses), so the models additionally correct for the anticoagulant.
